# Expression profile and prognostic values of Chromobox family members in human glioblastoma

**DOI:** 10.18632/aging.203912

**Published:** 2022-02-24

**Authors:** Juanni Li, Zhijie Xu, Lei Zhou, Kuan Hu

**Affiliations:** 1 Department of Pathology, Xiangya Hospital, Central South University, Changsha 410008, Hunan, China; 2 National Clinical Research Center for Geriatric Disorders, Xiangya Hospital, Central South University, Changsha 410008, Hunan, China; 3 Department of Anesthesiology, Third Xiangya Hospital of Central South University, Changsha 410008, Hunan, China; 4 Department of Hepatobiliary Surgery, Xiangya Hospital, Central South University, Changsha 410008, Hunan, China

**Keywords:** Chromobox (CBX), GBM, expression profiles, prognosis, immune infiltration

## Abstract

Glioblastoma (GBM) is the most common and deadly malignant primary brain tumor. Chromobox (CBX) family proteins are essential components of the epigenetic regulatory complex and are involved in the occurrence and development of various cancers. However, the roles of CBX members in GBM is little known. In this analysis, we synthesized several mainstream bioinformatics databases to comprehensively explore the expression profiles, prognostic implications, genetic alterations, immune infiltration, and potential biological functions of the CBXs in GBM, and cell experiments were also conducted to investigate the role of CBX8 in GBM. We found that the elevated mRNA expression of CBX2/3/5/8 and reduced mRNA expression of CBX6/7 were found in GBM. The protein levels of CBX2/3/5/8 were elevated in GBM tissues, whereas the protein levels of CBX6/7 showed no significant difference. The upregulated expression of CBX2/3/8 was found to be both correlated with the tumor grade and recurrent status. The overexpression of CBX3/8 and underexpression of CBX6 mRNA were associated with the poor prognosis. These findings suggested that CBX3 and CBX8 might be useful diagnostic and prognostic biomarkers in GBM. Further cell experiment results supported that CBX8 promoted the proliferation of glioma cells. Moreover, a high genetic alteration rate of CBXs (37%) was found in GBM and to varying degrees. The expression of CBXs was significantly related to the immune cells infiltration. CBX7 methylation level was significantly increased in GBM tissues. Our results may provide novel ideas to find potential prognostic markers and new therapeutic targets among CBX family members in glioblastoma.

## INTRODUCTION

Glioblastoma (GBM) is a deadly malignant primary brain tumor, constituting 54.9% of all gliomas [1, 2]. Even after various treatments including surgery, radiotherapy, and chemotherapy, the prognosis of patients with GBM is poor [3, 4]. In the past two decades, no significant change has been made in tumors progress and overall outcomes [5]. Thus, the identification of novel biomarkers for enhancing prognosis and individualized treatment effectively is of great significance.

So far, eight CBX family members (CBX1–8) have been found in mammalian cells [6, 7]. They are all participated in cell cycle regulation transcriptional repression, heterochromatin, and apoptosis [8, 9]. Depending on the basic molecular structure pattern of the CBXs, they can be split into the heterochromatin protein 1 (HP1) group (CBX1/3/5, also known as HP1*β*/*γ*/*α*) and the polycomb (PC) group (CBX2/4/6/7/8) [10, 11]. The CBX family had been reported to regulate the occurrence and development of a variety of tumors by limiting self-renewal and differentiation of tumor stem cells [12]. Emerging studies showed the dysregulated CBX members in multiple cancer types, including ovarian cancer [13], cervical cancer [14], pancreatic cancer [15], lung cancer [16], liver cancer [17], esophageal squamous cell carcinoma [18], breast cancer [19, 20] and gastric cancer [21] and so on. However, the precise functions of distinct CBX members in the development and progression of GBM remain elusive.

Therefore, in this work, we used multiple large public databases to explore the expression profiles, clinical relationships, prognostic implications, and immune infiltration of CBXs in GBM. We also investigated the predicted functions and pathways of the CBX family and their co-expressed genes. Our data highlighted the potential application value and mechanisms of CBXs in the prognosis and treatment of GBM.

## RESULTS

### Differential expression of CBXs in patients with GBM

**Figure 1:**
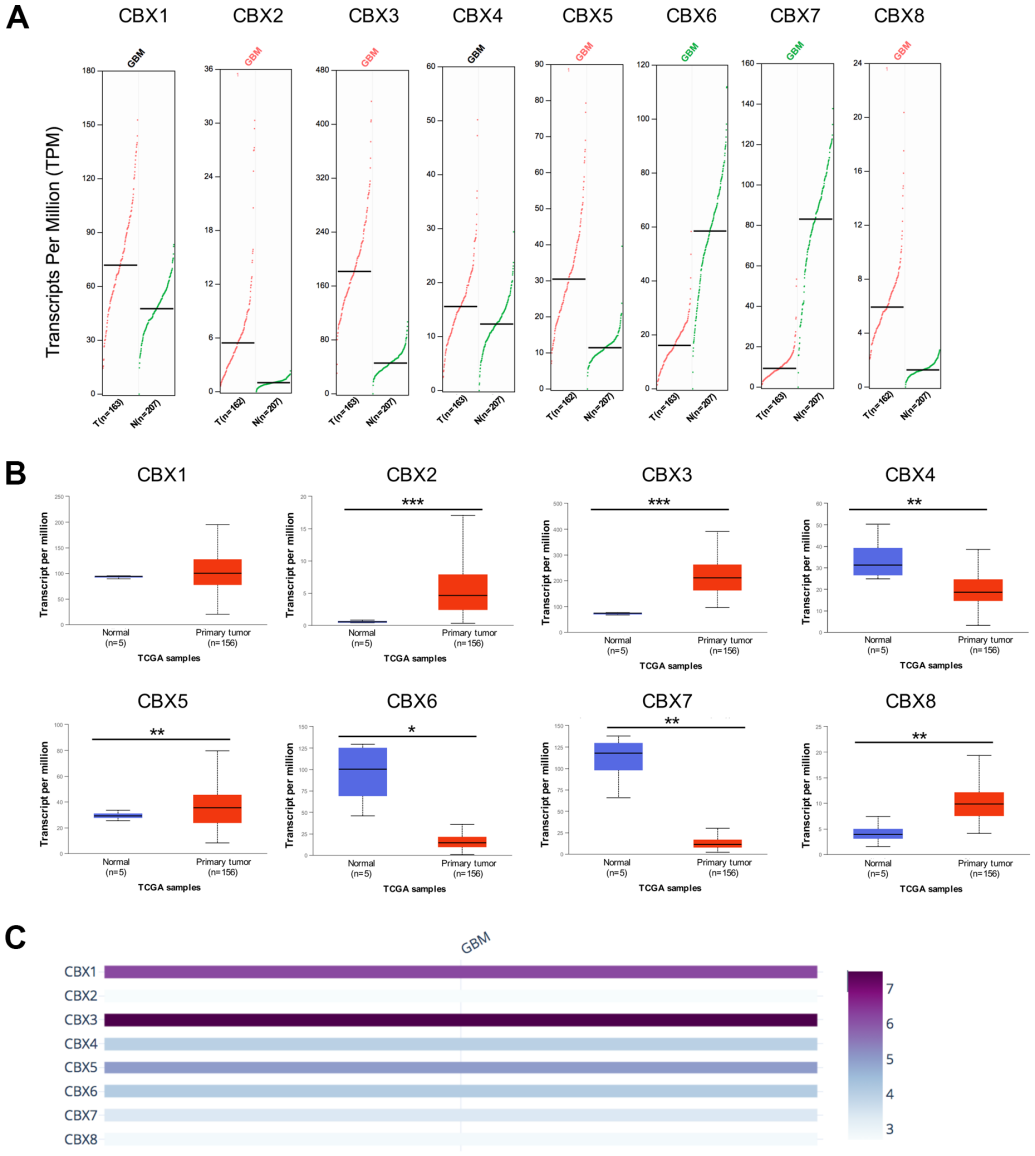
**mRNA expression levels of CBXs in GBM.** (**A**) mRNA expression levels of eight CBXs in GBM tissues and normal glial tissues from the GEPIA2. T: GBM tissues; N: normal tissues. (**B**) mRNA expression levels of eight CBXs in GBM tissues and normal glial tissues from the UALCAN. (**C**) The relative expression of eight CBX members in GBM. * *p* < 0.05, ** *p* < 0.01, *** *p* < 0.001.

To investigate the differential expression of distinct CBX family members in patients with GBM, mRNA expression and protein expression were analyzed with different databases. Firstly, according to the data acquired from GEPIA2, the mRNA expression levels of CBX2/3/5/8 were remarkably up-regulated in GBM tissues, while the mRNA expression levels of CBX6 and CBX7 were down-regulated in GBM tissues. However, the mRNA levels of CBX1 and CBX4 showed no difference between GBM tissues and normal glial tissues (Figure 1A). Then, the transcriptional levels of CBX family were further explored using UALCAN database and similar results were obtained. The results also showed elevated levels of CBX2/3/5/8, while reduced levels of CBX6 and CBX7 in GBM tissues vs. normal glial tissues. Besides, CBX4 was found to be down-regulated in GBM tissues (Figure 1B). Furthermore, we compared the relative expression of CBX members in GBM using GEPIA2 database and found that CBX3 had the highest and CBX2 had the lowest relative mRNA levels among all eight CBX members (Figure 1C).

**Figure 2:**
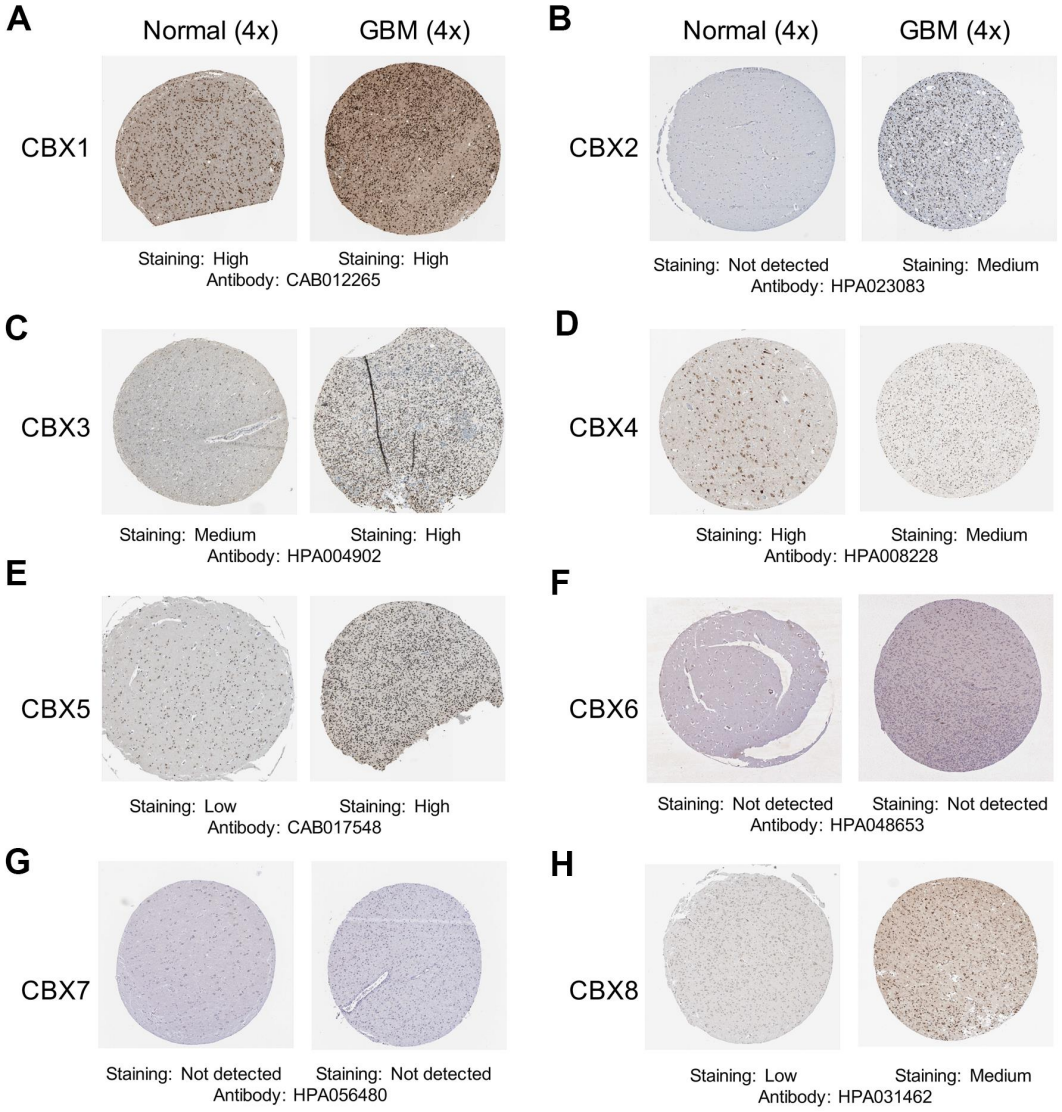
**Representative immunohistochemistry images of CBXs from The Human Protein Atlas.** (**A**–**H**) The protein expression profiles of eight CBXs in GBM tissues and normal glial tissues.

After determining the transcriptional expression of distinct CBX members in GBM, we then explored the immunohistochemistry (IHC) data about the protein expression patterns of CBX members in GBM using the Human Protein Atlas. The results exhibited that CBX2/3/5/8 protein expression levels were higher in GBM tissues (Figure 2B, 2C, 2E, 2H), while CBX4 protein level was lower in GBM tissues (Figure 2D). Additionally, the protein level of CBX1 showed no obvious difference between GBM tissues and paired normal glial tissues (Figure 2A). These results were consistent with our previous results on the CBXs mRNA expression. However, the protein levels of CBX6 and CBX7 were not observed in both GBM tissues and paired normal tissues and showed no significant difference (Figure 2F, 2G). This may be because the baseline expression of CBX6/7 in normal tissues was already very low, so it is hard to detect lower CBX6/7 expression in GBM tumor tissues.

### Clinicopathological parameters of CBXs in patients with GBM

**Figure 3:**
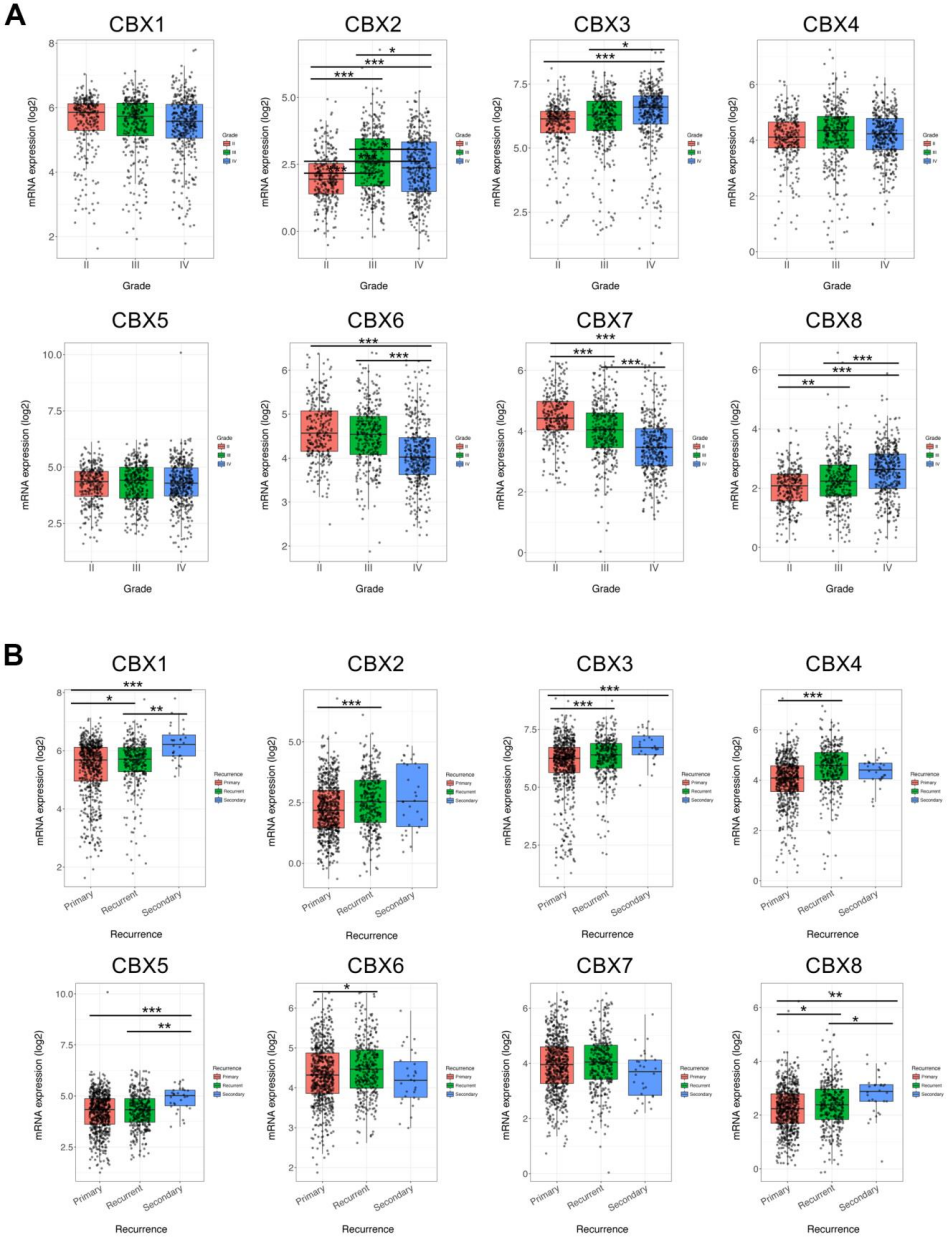
**Association of CBXs transcript levels with clinical pathology.** (**A**) Relationships between mRNA expression levels of eight CBX members and tumor grades of GBM. (**B**) Relationships between mRNA expression levels of eight CBX members and recurrent status of GBM. Analyses were conducted using GlioVis. * *p* < 0.05, ** *p* < 0.01, *** *p* < 0.001.

After a comprehensive analysis of each CBX member expression pattern, the relationships between the expression of differentially expressed CBX members and tumor grade and recurrence status in GBM were further investigated using the GlioVis database. As illustrated in Figure 3A, the mRNA levels of CBX2/3/6/7/8 were related to tumor grades, but the mRNA expressions of CBX1/4/5 were not markedly different. Statistically, the mRNA expression of CBX2/3/8 tended to be higher as tumor grade increased, whereas the mRNA expression of CBX6/7 tended to be lower with increasing tumor grade. The highest mRNA levels of CBX3/8 were observed in tumor grade IV, whereas the highest mRNA levels of CBX6/7 were detected in tumor grade II. Notably, CBX2 had a lower mRNA expression level in tumor grade IV compared to tumor grade III, and the highest mRNA levels of CBX2 were exhibited in tumor grade III.

Moreover, we then investigated the correlation between the expression of each CBX member and recurrence status in GBM. As shown in Figure 3B, except for CBX7, the mRNA levels of CBX1/2/3/4/5/6//8 was all significantly associated with tumor recurrence status. Those CBX members had higher expression levels in recurrent tumors compared to primary tumors. The above findings were almost consistent with our previous results on CBXs expression, except for CBX4/6 whose expression levels were lower in GBM tumor tissues. Summarily, based on these findings on the expression profiles of CBX members and the relationships between CBXs expression and clinicopathological parameters, CBX2/3/8 might play critical roles in the tumorigenesis and recurrence of GBM.

### Prognostic value of mRNA level of CBXs in patients with GBM

**Figure 4:**
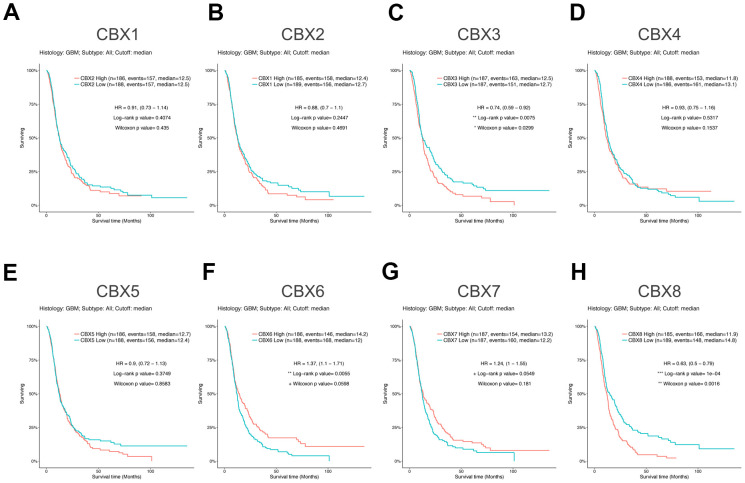
**Prognostic value of CBXs transcript levels in GBM.** (**A**–**H**) Relationships between mRNA expression levels of eight CBX members and the prognosis of patients with GBM. Analyses were conducted using the GlioVis.

To investigate the worth of the CBXs in the progression of GBM, we applied the GlioVis database to analyze correlations between differentially expressed CBXs and clinical outcomes. As shown in Figure 4, overexpression of CBX3/8 was correlated to a short survival time, whereas higher expression of CBX6 was significantly correlated to a longer survival time. In addition, the rest of the CBX members did not seem to be associated with survival time in patients with GBM. Thus, the mRNA expression levels of CBX3/6/8 were obviously associated with the prognosis of GBM and could potentially be applied as a useful predictive biomarker. Conclusively, based on our comprehensive analysis of the expression, clinicopathological parameters, and prognostic values of eight CBX members, CBX3 and CBX8 were identified and might play pivotal roles in the tumorigenesis and progression of glioblastoma.

### CBX8 promoted the proliferation of glioma cells

**Figure 5:**
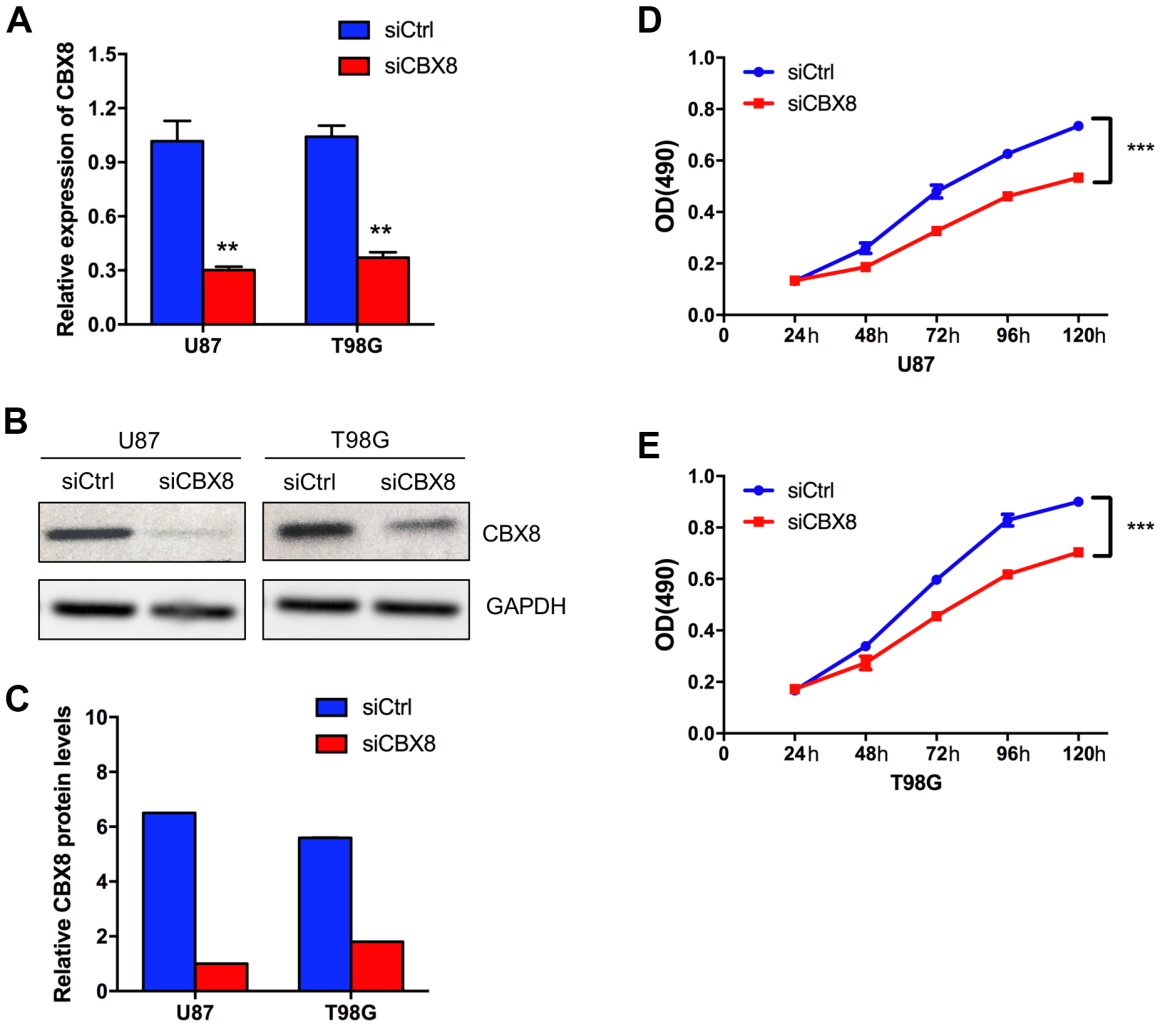
**CBX8 promoted the proliferation of glioma cells.** (**A**) The mRNA levels of CBX8 in U87 and T98G cells transfected with siCtrl and siCBX8. (**B**, **C**) The protein levels of CBX8 in U87 and T98G cells transfected with siCtrl and siCBX8. (**D**, **E**) The CCK-8 assay was applied to measure the effect of siCBX8 on the growth of U87 and T98G cells. ** *p* < 0.01, *** *p* < 0.001.

Several works have explored the function of CBX8 in GBM. Whereas the role of CBX8 in GBM has not been fully elucidated. To investigate the role of CBX8 in glioma cell growth, we used a CCK-8 assay to prove the effect of knockdown of CBX8 on cell proliferation. First, we transfected siCBX8 or siCtrl into two human glioma cell lines including U87 cells and T98G cells. The inhibitory effect on CBX8 expression was detected by qPCR and Western Blot. As shown in Figure 5A–5C, compared with siCtrl group, CBX8 knockdown would substantially inhibit the expression of CBX8 with siCBX8. Then, using the CCK-8 assay, we found that CBX8 knockdown suppressed the proliferation of glioma cells with siCBX8 (Figure 5D, 5E). These findings suggested that CBX8 promoted the proliferation of glioma cells.

### Genetic alteration and functional analysis of CBXs in patients with GBM

**Figure 6:**
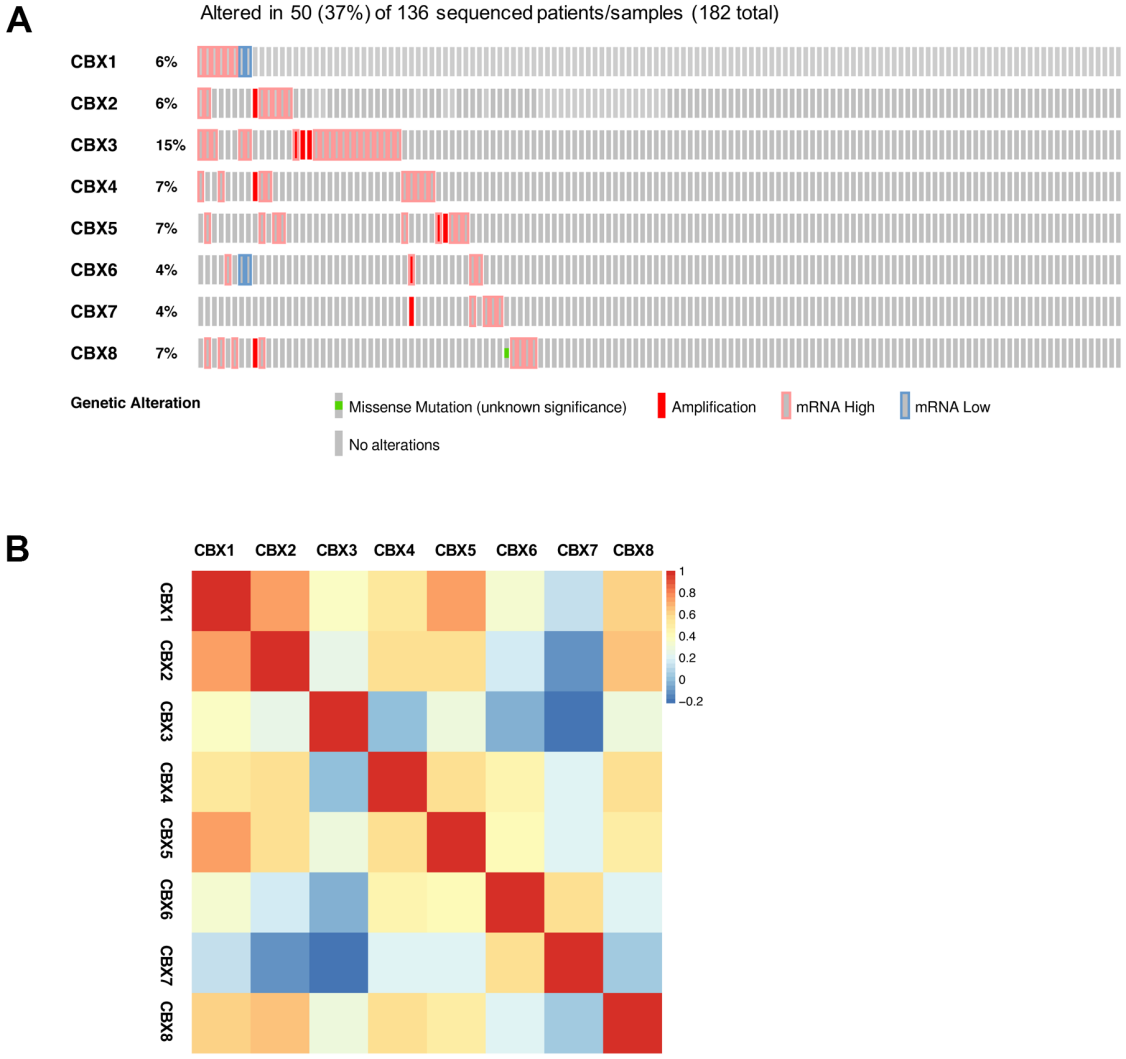
**Genetic alterations and correlation analysis of CBXs in GBM.** (**A**) summary of eight CBX member alteration in GBM (cBioPortal). (**B**) Correction of eight CBX members with each other (GEPIA2).

We explored the genetic alterations of CBXs in GBM using the cBioPortal. As shown in Figure 6A, the CBXs gene was altered in 50 samples from 136 glioblastoma patients, accounting for a 37% alteration rate. According to the TCGA Firehose Legacy dataset, the percentages of genetic alterations in CBX1-8 were 6, 6, 15, 7, 7, 4, 4, and 7%, respectively, in GBM (Figure 6A). Next, we evaluated the correlation of distinct CBX members with each other through analyzing their mRNA expression. As shown in Figure 6B, several significant positive correlations were found: CBX1 with CBX2, CBX5, and CBX8; CBX2 with CBX4 and CBX8; CBX6 with CBX7. Besides, the CBXs with significant negative correlation was shown: CBX3 with CBX7 (Figure 6B).

**Figure 7:**
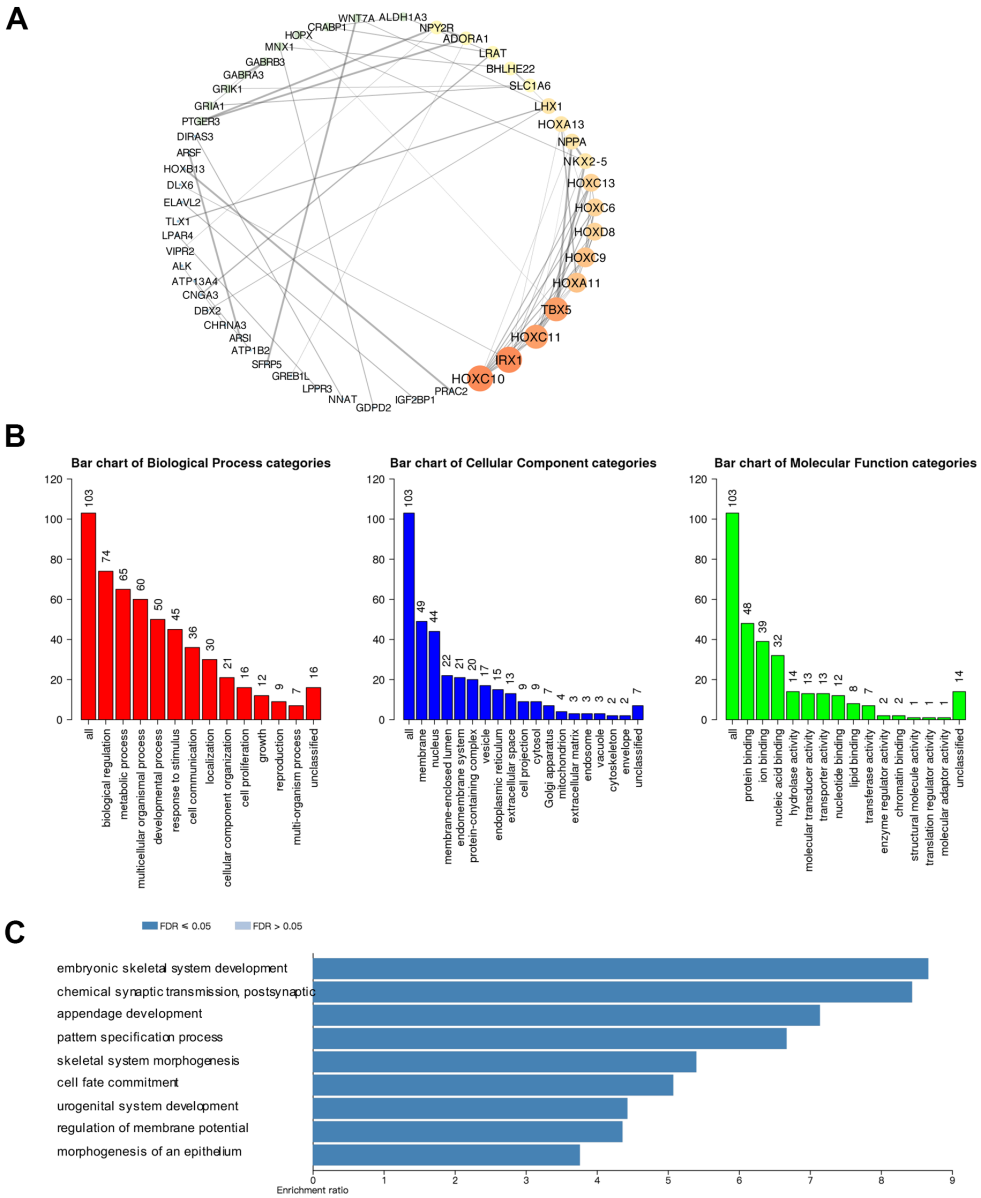
**Predicted protein-protein interactions, functions, and pathways of CBXs and their co-expressed genes in GBM.** (**A**) Protein-protein interaction (PPI) enrichment analysis of the 103 co-expressed genes of CBXs was constructed in cBioPortal and Cytoscape. The color of nodes: low values to bright colors; high values to dark colors. The thickness of edges: low values to fine edges; high values to thick edges. (**B**) Gene Ontology (GO) functional enrichment analysis of CBXs co-expressed genes (WebGestalt), including biological process, cellular components, and molecular functions. (**C**) Kyoto Encyclopedia of Genes and Genome (KEGG) analysis of CBXs co-expressed genes (WebGestalt).

Furthermore, we explored the function of the CBXs in GBM patients. We applied the cBioPortal to obtain 103 co-expressed genes that were most relevant with CBXs in GBM and used the Cytoscape to construct a protein-protein interaction (PPI) network (Supplementary Table 2). As shown in Figure 7A, HOXC10, IRX1, HOXC11, and TBX5 were primarily related to the function of the CBXs in GBM (Figure 7A). Then, the functions of the CBX members and their frequently altered neighbor genes and the molecular mechanisms by which CBX family regulated glioblastoma were subsequently analyzed in the WebGestalt. Gene Ontology (GO) functional annotation included biological process (BP), cellular component (CC), and molecular function (MP), and the results showed that CBX proteins were primarily associated with biological regulation and metabolic process in BP. As for CC, the CBX proteins were mainly associated with the membrane and nucleus. Besides, the CBXs influenced MF through protein binding and ion binding (Figure 7B). As shown in Figure 7C, the KEGG pathways involved in these co-expressed genes were subsequently explored and nine pathways were of significance. We discovered that these co-expressed genes were primarily involved in signaling pathways of embryonic skeletal system development and chemical synaptic transmission, postsynaptic (Figure 7C).

### Immune cell infiltration of CBXs in patients with GBM

**Figure 8:**
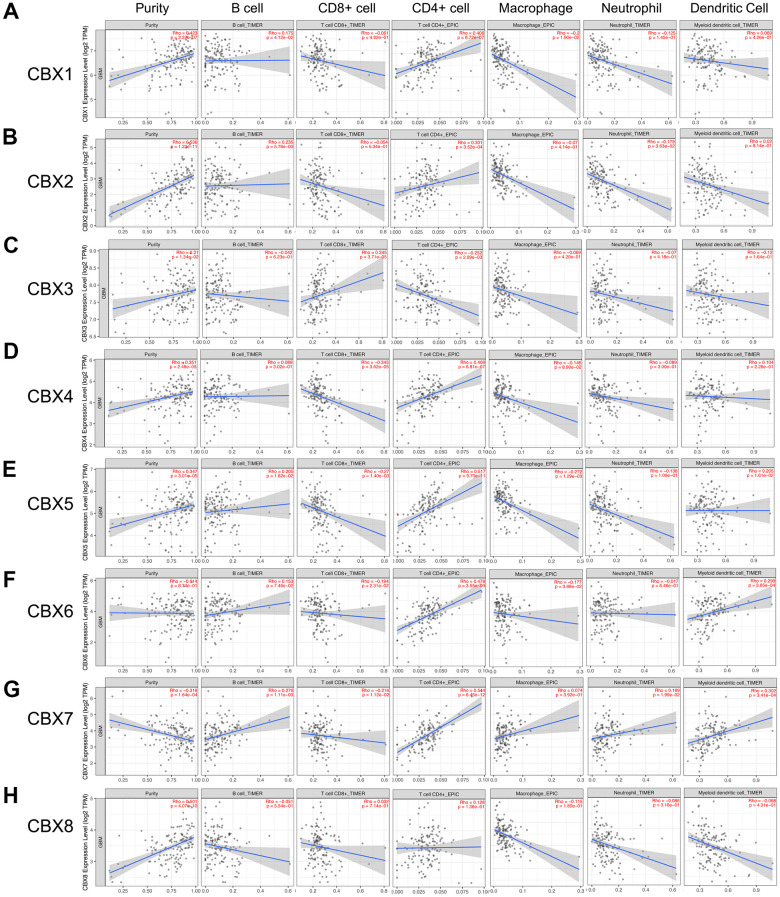
**Correlations between CBXs and immune cell infiltration (TIMER2.0).** (**A**–**H**) The effects of eight CBX members on the infiltration of six immune cells including B cells, CD8+ T cells, CD4+ T cells, macrophages, neutrophils, and dendritic cells. Each dot in the scatter plots represented a single tumor sample.

Emerging evidence supported that immune cell levels were correlated with tumorigenesis and progression in multiple tumor types [7, 22]. In this analysis, through the TIMER2.0, we investigated the correlations between different CBX members and immune cell infiltration levels in GBM. As shown in Figure 8 and Supplementary Figure 1, CBX1/2 was both positively related to B cells and CD4+ T cells, CBX1 was also negatively related to macrophages and CBX2 was negatively related to neutrophils (Figure 8A, 8B). Besides, CBX3/4 were both related to CD8+ and CD4+ T cells: CBX3 was positively related to CD8+ T cells and was negatively associated with CD4+ T cells, whereas CBX4 had the opposite trend (Figure 8C, 8D). In addition, CBX5/6 were both positively related to CD4+ T cells and dendritic cells and were negatively related to CD8+ T cells and macrophages, CBX5 was also positively correlated with B cells (Figure 8E, 8F).

Moreover, CBX7 was negatively related to CD8+ T cells and was positively related to the infiltration of four immune cells. These cells included B cells, dendritic cells, neutrophils, and CD4+ T cells (Figure 8G). However, CBX8 showed no correlations with the infiltration of these immune cells (Figure 8H).

### Methylation expression levels of CBXs in patients with GBM

DNA methylation has been found to be negatively correlated to the expression level of genes in various cancers [23, 24]. Here, we used the DiseaseMeth database to investigate the DNA methylation levels between GBM tissues and paired normal glial tissues. As shown in Supplementary Figure 2, the methylation levels of CBX7 were increased in GBM tissues. Our previous mRNA expression data showed the down-regulated expression of CBX7 in GBM tissues, and its reduced expression levels in GBM tissues might be because of its high methylation levels. However, except for CBX7, the methylation levels of other CBXs did not have a significant difference between GBM tumor tissues and paired normal glial tissues (Supplementary Figure 2), and there may be other correlated factors affecting their expression levels, such as posttranscriptional regulation, genetic alterations, and so on.

## DISCUSSION

Glioblastoma is an aggressive primary malignant brain tumor, accounting for about 54.9% of all gliomas [25–27]. Emerging evidence indicated that epigenetic alterations were frequently detected in various cancers and played a pivotal role in tumorigenesis and progression of these cancers [28]. Epigenetic alterations contained aberrant histone modifications, abnormal DNA methylation, and regulated several non-coding RNAs expression [29]. Moreover, Polycomb group (PcG) proteins, as a class of epigenetic regulators, played a critical role in Polycomb inhibitory complexes (including PRC1 and PRC2) and were involved in the regulation of aging, cell proliferation, and tumorigenesis [30]. Hence, CBX members, which serve as the normative component of PRC1, may be related to epigenetic regulation through targeting PRC1 to chromatin [31]. To date, several CBX members were found to play important roles in several tumor types [18, 32], but the distinct roles of CBXs in GBM have not been fully elucidated. This study is the first time to comprehensively analyze their expression profiles, and explore their associations with the clinical features, prognostic implications, immune cell infiltration, methylation, and potential functions in GBM by using bioinformatics. We hoped that our work would help to improve early diagnosis and new therapeutic options in GBM patients.

For the first time, the transcriptional and protein expression levels of CBXs were summarized in GBM by using different databases. According to the data obtained from both GEPIA2 and UALCAN, we found elevated transcriptional expression levels of CBX2/3/5/8, while reducing levels of CBX6/7 in GBM tissues vs. normal tissues. Then, the protein levels of CBXs were further evaluated and the elevated protein expression levels of CBX2/3/5/8 were identified which was consistent with their mRNA expression pattern. However, the protein levels of CBX6/7 were not detected in both GBM tissues and paired normal tissues and showed no significant difference. Based on these findings above, CBX2/3/5/8 might play critical roles in the tumorigenesis and progression of GBM. Moreover, the relationships between the CBXs expression and tumor grade and recurrence status in GBM were further explored. The results exhibited that the mRNA expression of CBX2/3/8 tended to be higher as tumor grade increased, whereas the mRNA expression of CBX6/7 tended to be lower with increasing tumor grade. In addition, except for CBX7, the overexpression levels of CBX1/2/3/4/5/6//8 mRNA were detected in recurrent tumors compared to primary GBM tumors. Furthermore, we investigated the prognostic implications of the CBXs in the progression of GBM and found that overexpression of CBX3/8 mRNA, as well as underexpression of CBX6 mRNA, was significantly associated with shorter survival time. Summarily, based on our comprehensive analysis about the expression, clinicopathological parameters, and prognostic values of different CBXs, only CBX3 and CBX8, which got consistent results on all these aspects, were identified as likely to take a critical part in the development of glioblastoma.

Aberrant expression of CBX3 has been detected in several cancer types such as pancreatic cancer, osteosarcoma, colorectal cancer, breast cancer, and liver cancer. CBX3 has been found to be highly expressed and promote aerobic glycolysis by suppressing FBP1 in pancreatic cancer [33]. In patients with osteosarcoma, CBX3 is overexpressed which is associated with larger tumor sizes, higher metastasis rates, and poor prognosis [34]. In colorectal cancer, Liu et al. found that miR-30a could target CBX3 to inhibit colorectal cancer cell growth by a xenograft mouse model [35]. In breast cancer and liver cancer, CBX3 was identified to be highly expressed, and its overexpression could enhance tumor cell proliferation and predict a poor prognosis [36, 37]. In our analysis, CBX3 was found to be highly expressed in GBM tissues, and its expression was obviously related to several clinicopathological parameters and poor prognosis in GBM. These outcomes highlighted that CBX3 may function as an oncogene and play a critical role in the tumorigenesis and development of GBM.

Existing evidences showed that CBX8 could regulate cell cycle progression, senescence, and differentiation in a variety of cancer types. In bladder cancer, CBX8 was found to inhibit the p53 pathway to enhance tumor cell proliferation, and its overexpression was positively associated with a poor prognosis [38]. In esophageal squamous cell carcinoma, Wang et al. found that CBX8 could promote tumor cell proliferation but inhibit cell metastasis by inhibiting Snail, and acted as a contradictory role [39]. Further verification was needed in the future to confirm its functionality. In hepatocellular carcinoma, CBX8 has been reported to exhibit oncogenic activity via AKT/β-Catenin activation [40], and its overexpression was inversely correlated with patient survival time [17]. Yang et al. reported that down-regulated CBX8 could induce tumor cell apoptosis in colorectal cancer cells [41]. To date, there is no precise analysis exhibited the exact role of CBX8 in GBM. In this analysis, we exhibited that the expression levels of CBX8 were increased in GBM and positively correlated with higher tumor grade and recurrent status. Its overexpression levels represented a shorter survival time in patients with GBM. Besides, through CCK8 assay and transient transfection assay, we found that CBX8 promoted the proliferation of glioma cells. These data suggested that CBX8 might be a potential biomarker for glioblastoma, and guide clinical treatment.

Immune infiltration has become an increasingly popular topic and took a significant part in the progression and recurrence of various cancers [42, 43]. Immune cells have been reported to act as either tumor-suppressing or tumor-promoting activities. They were deemed to be a pivotal determinant of both the response to immunotherapy and the patient's clinical outcome [44–46]. In this study, we found that except for CBX8, the expression of other seven CBX family members was obviously related to six immune cells infiltration, suggesting that distinct CBX family member might reveal the immune status in GBM patients in addition to the prognosis. Especially for CD4 + T cells, the expression of CBX1-7 members were all related to its infiltration: the expression of CBX3 was negatively related to its infiltration, while the expression of CBX1/2/4/5/6/7 was positively related to its infiltration, suggesting that CD4 + T cells may play an essential role in the CBX family’s influence on the immune status of GBM. Our findings might provide some useful immunization information to help design the new immunotherapy and improve patient prognosis.

However, we note some limitations in this study. Most of our data analyzed in this work was taken from online databases, further study consisting of clinical studies or more cell experiments would be required to validate our discoveries *in vivo* and *in vitro*.

## CONCLUSIONS

In conclusion, we first systematically explored CBXs expression, its correlation with the clinical features, prognostic implications, immune cell infiltration, methylation, and potential functions in GBM, opening broad prospects for the potential of CBX members as prognostic markers and treatment targets of GBM. The elevated transcriptional expression of CBX2/3/5/8 and reduced expression of CBX6/7 were detected in GBM. The protein levels of CBX2/3/5/8 were higher while that of CBX6/7 showed no significant difference in GBM tissues. In addition, the upregulated expression of CBX2/3/8 and downregulation of CBX6/7 were discovered to be associated with the tumor grade. Except for CBX7, the overexpression levels of CBX1/2/3/4/5/6//8 mRNA were detected in recurrent tumors compared to primary GBM tumors. Moreover, overexpression of CBX3/8 mRNA, as well as underexpression of CBX6 mRNA, was related to a shorter survival time. Moreover, through CCK8 assay and transient transfection assay, we found that CBX8 promoted the proliferation of glioma cells. Furthermore, a high genetic alteration rate of CBXs (37%) was found in GBM and to varying degrees. The mRNA expression of CBXs was observed to be related to the infiltration of six immune cells, especially CD4 + T cells. The methylation levels of CBX7 were significantly increased in GBM which was consistent with its mRNA expression pattern. Our findings may provide new ideas to identify novel diagnostic and prognostic biomarkers among CBX members in glioblastoma.

## MATERIALS AND METHODS

### GEPIA2

GEPIA2 is an interactive web server using standard processing pipelines and analyzing the RNA sequencing expression data of thousands of cancer tissues and normal tissues from the TCGA and GTEx projects [47, 48]. In this work, we applied GEPIA2 to analyze the mRNA levels of CBXs between GBM tumor tissues and normal glial tissues. The *p*-value was generated by the Student’s t-test. |Log2FC| >1 and *p*<0.01 were significant. Besides, we also used the “correlation analysis” model of GEPIA2 to evaluate the correlation of the CBXs with each other. The databases applied in this work were summarized in Supplementary Table 1.

### UALCAN

UALCAN is a comprehensive online platform containing the clinical data and level 3 RNA-seq data from TCGA database [49]. In this work, UALCAN was used to compare the transcriptional expression levels of distinct CBX members between GBM tissues and normal glial tissues.

### The Human Protein Atlas

The Human Protein Atlas is an interactive online tool including transcriptome profiles and immunohistochemistry profiles for nearly 20 cancer types and thousands of patients [50]. In our analysis, we used immunohistochemical images downloaded from this database to compare the protein expression levels of distinct CBX members between GBM tumor tissues and normal glial tissues and evaluated their protein expression patterns in depth.

### GlioVis

GlioVis is a powerful web application for the visualization and analysis of brain tumor expression datasets [51]. In our work, we used GlioVis to establish the correlation between mRNA expression levels of different CBX members and tumor grade and recurrent status of GBM. In addition, we also used this database to analyze the prognostic implications of CBXs mRNA expression in GBM. The Chinese Glioma Genome Atlas (CGGA) database containing over 600 glioma samples was chosen.

### cBioPortal

cBioPortal is a comprehensive publicly web tool that could provide researchers with visual and multidimensional tumor genomics data [52, 53]. In our study, the Glioblastoma Multiforme (TCGA, Firehose Legacy) dataset containing 604 samples was selected to analyze the CBXs in cBioPortal. We evaluated the genetic alterations and transcriptional expression of CBXs.

### Cytoscape

Cytoscape is an open source software project for integrating models of biomolecular interaction networks [54]. In this analysis, we used Cytoscape to execute functional integration on 103 co-expressed genes of the CBXs obtained from the cBioPortal (Supplementary Table 2). The size of nodes represented the degree values of interacted proteins. The higher the degree, the larger the nodes.

### WebGestalt

WebGestalt is a comprehensive and interactive gene set enrichment analysis toolkit that was used for gene lists interpretation. These gene lists can be derived from large scale -omics studies [55]. In our work, the KEGG pathway and GO enrichment were conducted by using this tool.

### TIMER2.0

TIMER2.0 is a public web tool that could be used to analyze the relationships between cancers and immunity [56]. In this work, “Gene module” was applied and scatterplots were obtained to analyze the correlation between the expression levels of eight CBX members and the infiltrating of six immune cells in GBM.

### DiseaseMeth2.0

DiseaseMeth2.0 is a kind of human disease methylation database that could present the most complete annotation and collection of abnormal DNA methylation in various human diseases, especially cancers [57, 58]. In our study, the DNA methylation status of CBX members between GBM tumor tissues and normal glial tissues was evaluated by this database.

### Cells

The glioma cell lines U87 and T98G were cultured as previously described [59].

### Transient transfection

For transient transfections assay, siRNAs for CBX8 were purchased from Ribobio (SIGS0002853-4, Ribobio), and the transfection reagent used here was Lipofectamine 3000 reagent (Invitrogen). After the designated transfection times, the U87 and T98G cells were gathered and studied.

### RNA extraction and qRT-PCR analyses

The process of RNA extraction and qRT-PCR was the same as previously described [13]. GAPDH was an internal control for quantification. The primer sequences of CBX8 were F: 5′ACGGAAAGGACGCATGGAAT3′; R: 5′CTTGGGTCCACGCTTTTTGG 3′.

### Western blot

Protein samples were prepared and separated by SDS-PAGE gels, transferred onto PVDF membrane, and blocked with 5% non-fat milk. Then, blots were hybridized with the following primary antibodies: CBX8 (ab259849, Abcam), GAPDH (sc-47724, Santa Cruz Biotechnology). The detection of protein levels was conducted using Image Lab software (Bio-Rad, CA, USA).

### CCK8 assay

The Cell Counting Kit-8 (Dojindo, Japan) was applied to detect cell growth according to the manufacturer’s instructions.

### Statistical analyses

Statistical analyses were performed with SPSS, version 18.0 (Chicago, USA). All experiments were performed in at least triplicate. The difference among groups was determined by Student’s t-test. P < 0.05 was statistically significant.

## Supplementary Material

Supplementary Figures

Supplementary Tables
