# Supplementary material for: Expression profile and prognostic values of Chromobox family members in human glioblastoma
Source: Aging (Albany NY). 2022 Feb 24;14(4):1910–31. doi: 10.18632/aging.203912 (PMC8908931; doi:10.18632/aging.203912)
Supplement: Supplementary Figures [file aging-14-203912-s001.pdf]

SUPPLEMENTARY FIGURES

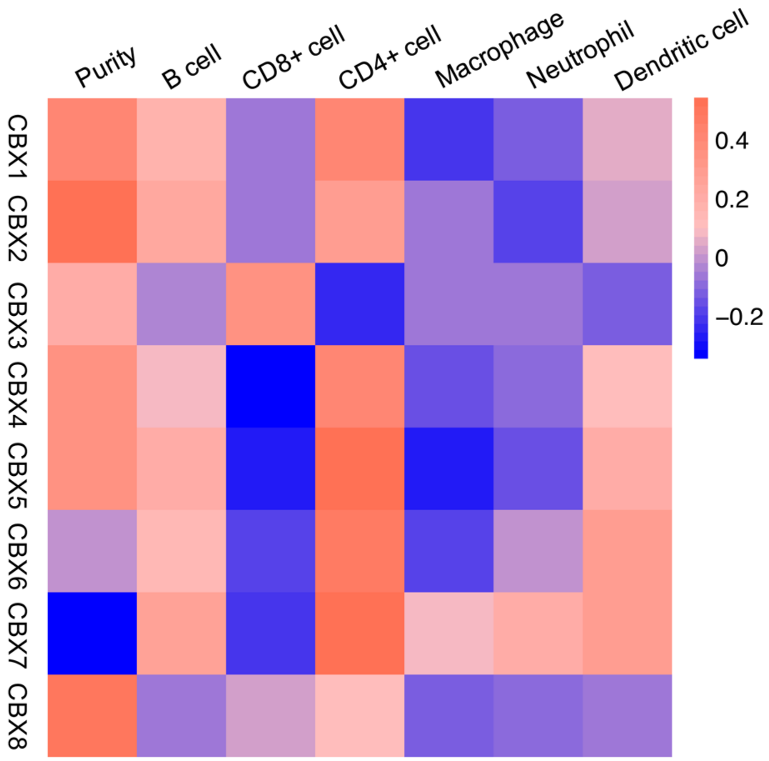

Supplementary Figure 1. The heat map showed the correlations between CBXs and immune cell infiltration.

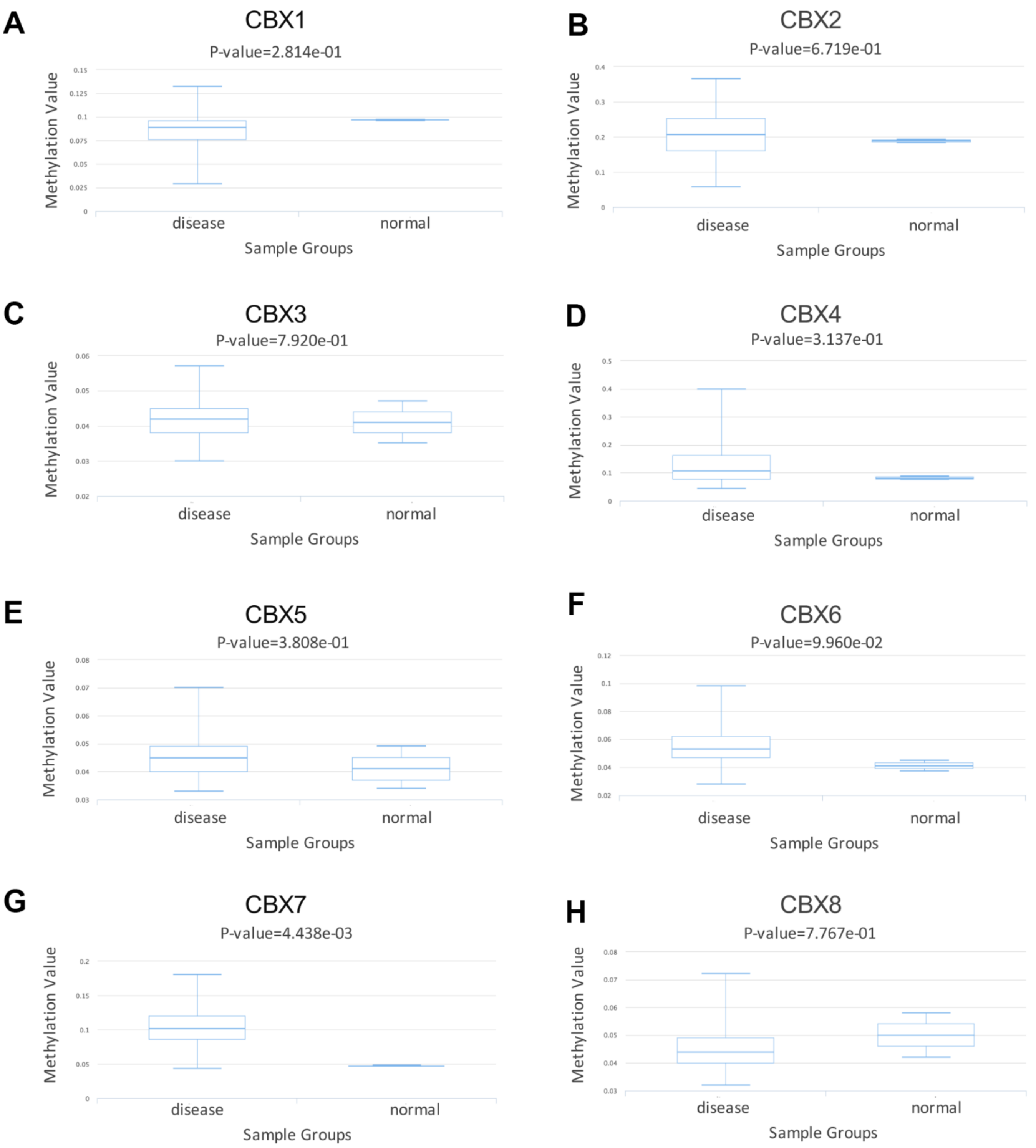

Supplementary Figure 2. DNA methylation levels of CBXs in GBM (DiseaseMeth). (**A**–**H**) The DNA methylation values of eight CBX members in GBM tissues and normal tissues.
