# Supplementary material for: Expression profile and prognostic values of Chromobox family members in human glioblastoma
Source: Aging (Albany NY). 2022 Feb 24;14(4):1910–31. doi: 10.18632/aging.203912 (PMC8908931; doi:10.18632/aging.203912)
Supplement: Supplementary Tables [file aging-14-203912-s002.pdf]

SUPPLEMENTARY TABLES

Supplementary Table 1. A series of bioinformatics databases for analyzing the role of the CBX family in GBM.

Databases	Authors	Samples	Homepage links
GEPIA2	Tang Z. et al.	Tissues	http://gepia2.cancer-pku.cn/
UALCAN	Chandrashekar DS	Tissues	http://ualcan.path.uab.edu/index.html
The Human Protein Atlas	Anna Asplund. et al.	Tissues	https://www.proteinatlas.org/
GlioVis	Bowman RL. et al.	Tissues	http://gliovis.bioinfo.cnio.es
cBioPortal	Cerami E. et al.	Tissues	http://www.cbioportal.org/
Cytoscape	Doncheva NT et al.	-	-
WebGestalt	Liao Y. et al.	-	http://webgestalt.org/
TIMER2.0	Li T. et al.	Tissues	http://timer.cistrome.org/
DiseaseMeth2.0	Xiong Y. et al.	Tissues	http://bio-bigdata.hrbmu.edu.cn/diseasemeth/

Supplementary Table 2. The CBX family associated co-expressed genes in GBM.

Gene	Log ratio	p-Value	Expression
NPNT	-1.28	9.99E-05	Unaltered group
ADORA1	-0.85	1.08E-04	Unaltered group
ATP1B2	-0.82	1.58E-04	Unaltered group
EBF3	1.61	2.18E-04	Altered group
BNC1	0.83	2.52E-04	Altered group
MLC1	-0.87	2.94E-04	Unaltered group
PCDHGB7	-1.62	2.95E-04	Unaltered group
FGFR3	-1.07	4.93E-04	Unaltered group
GRIK1	-1.08	7.49E-04	Unaltered group
PLA2G5	-1.36	7.85E-04	Unaltered group
ESR2	-0.86	8.00E-04	Unaltered group
TMEM98	0.81	1.01E-03	Altered group
CDH4	-0.92	1.05E-03	Unaltered group
SLC4A11	-1	1.17E-03	Unaltered group
DMRTA2	-1.24	1.72E-03	Unaltered group
CMYA5	-0.83	2.13E-03	Unaltered group
ARHGAP28	1	2.17E-03	Altered group
FBLN7	-0.82	2.20E-03	Unaltered group
LRAT	-1.04	2.27E-03	Unaltered group
EPCAM	0.84	2.41E-03	Altered group
GDPD2	-0.83	2.41E-03	Unaltered group
HOXB13	1.63	2.46E-03	Altered group
ACSS3	-0.86	2.95E-03	Unaltered group
ALK	-0.89	3.04E-03	Unaltered group
USP43	1.07	3.15E-03	Altered group
PDE10A	0.84	3.20E-03	Altered group
ARSF	-1.09	3.38E-03	Unaltered group
ADAMTS17	0.83	3.41E-03	Altered group
FAM181A	-0.81	3.68E-03	Unaltered group
CNGA3	-0.95	4.88E-03	Unaltered group
TBX5	1.62	4.89E-03	Altered group
DIRAS3	-0.84	5.05E-03	Unaltered group
LPAR4	0.92	5.14E-03	Altered group
PTGER3	0.86	5.55E-03	Altered group
DUSP9	0.84	5.62E-03	Altered group
ARSI	-0.85	5.79E-03	Unaltered group
TFCP2L1	-0.91	5.83E-03	Unaltered group
CLGN	1.08	6.14E-03	Altered group
DLX6	1.23	6.42E-03	Altered group
GREB1L	0.86	7.24E-03	Altered group
PRAC2	1.12	7.42E-03	Altered group
CRABP1	1.45	7.61E-03	Altered group
ATP13A4	-0.82	7.87E-03	Unaltered group
HOXD8	0.98	7.90E-03	Altered group
HOXA11	0.95	8.04E-03	Altered group
WNT7A	-0.9	8.24E-03	Unaltered group
CHRNA3	0.87	8.25E-03	Altered group
NXPH2	0.93	8.28E-03	Altered group
ELAVL2	1.03	8.95E-03	Altered group
NKX2-5	-1.09	9.57E-03	Unaltered group
NNAT	1.35	9.88E-03	Altered group
HS3ST3B1	-0.84	0.01	Unaltered group
HOXC6	0.86	0.0102	Altered group

ELOVL2	-0.83	0.0111	Unaltered group
SAMD11	0.85	0.0112	Altered group
ECEL1	1.12	0.0117	Altered group
SLC1A6	1.11	0.0122	Altered group
FEV	0.93	0.0127	Altered group
CYP27C1	0.9	0.0129	Altered group
DMRT2	0.92	0.0131	Altered group
PAK5	1.11	0.0131	Altered group
GRIA1	-0.85	0.0136	Unaltered group
TMEM229A	-0.85	0.0137	Unaltered group
ALDH1A3	1.14	0.0138	Altered group
DBX2	-0.81	0.014	Unaltered group
CHRDL2	-0.97	0.0156	Unaltered group
C6ORF15	-1.23	0.0162	Unaltered group
MNX1	1	0.0172	Altered group
TLX1	1.09	0.0176	Altered group
LHX1	0.85	0.0178	Altered group
OTX1	-0.86	0.018	Unaltered group
CPNE4	-1.13	0.018	Unaltered group
CAMKV	0.9	0.0184	Altered group
DCT	0.82	0.0186	Altered group
HOXA13	0.87	0.0187	Altered group
HOPX	-0.85	0.0187	Unaltered group
BARX1	0.87	0.0202	Altered group
VIPR2	1.19	0.0203	Altered group
BHLHE22	0.91	0.0207	Altered group
DYDC2	0.83	0.0208	Altered group
SHISA2	0.87	0.0223	Altered group
HOXC13	1.14	0.0227	Altered group
MOXD1	-0.86	0.023	Unaltered group
IGF2BP1	0.83	0.0239	Altered group
HMX1	1.2	0.0247	Altered group
SFRP5	0.92	0.0251	Altered group
CDR1	0.91	0.0276	Altered group
APCDD1L	-0.89	0.0285	Unaltered group
SLC44A5	0.91	0.0287	Altered group
ABCA13	-0.81	0.0293	Unaltered group
IRX1	1.38	0.0301	Altered group
GABRB3	0.88	0.0303	Altered group
NPPA	0.93	0.0314	Altered group
PLPPR3	0.98	0.0321	Altered group
HOXC9	0.86	0.0325	Altered group
CNPY1	0.84	0.0345	Altered group
KLRC4	0.83	0.035	Altered group
NPY2R	-0.9	0.0383	Unaltered group
HOXC11	0.94	0.0384	Altered group
JCHAIN	0.99	0.04	Altered group
HOXC10	1	0.0408	Altered group
CXCL14	-0.83	0.0414	Unaltered group
GABRA3	0.81	0.0468	Altered group
PCDHGA10	-0.91	0.049	Unaltered group
